# Association between plasma glucose-dependent insulinotropic polypeptide and active adiponectin in normoglycemic women

**DOI:** 10.1530/EC-25-0804

**Published:** 2026-01-13

**Authors:** Isidora Salvatierra, Javier Parada, Rodrigo Cataldo, José Eduardo Galgani, Gigliola Alberti, Idoia Labayen, José L Santos

**Affiliations:** ^1^ Departamento de Nutrición, Diabetes y Metabolismo, Escuela de Medicina, Facultad de Medicina, Pontificia Universidad Católica de Chile, Santiago, Chile; ^2^ Instituto de Ciencia y Tecnología de los Alimentos (ICYTAL), Facultad de Ciencias Agrarias y Alimentarias, Universidad Austral de Chile, Valdivia, Chile; ^3^ Clinical Research Center, Department of Clinical Sciences in Malmö, Lund University Diabetes Centre, Lund, Sweden; ^4^ Departamento de Nutrición y Dietética, Escuela de Ciencias de la Salud, Facultad de Medicina, Pontificia Universidad Católica de Chile, Santiago, Chile; ^5^ Department of Pediatric Gastroenterology and Nutrition, Division of Pediatrics, School of Medicine, Pontificia Universidad Católica de Chile, Santiago, Chile; ^6^ Department of Health Sciences, Institute for Sustainability and Food Chain Innovation (IS-FOOD), Public University of Navarre, Pamplona, Spain; ^7^ IdiSNA, Navarra Institute for Health Research (IdiSNA), Pamplona, Spain; ^8^ CIBER de Fisiopatología de la Obesidad y Nutrición (CIBEROBN), Instituto de Salud Carlos III, Madrid, Spain

**Keywords:** incretins, adiponectin, high molecular weight adiponectin, GIP, adipokines

## Abstract

**Background/objectives:**

Glucose-dependent insulinotropic polypeptide (GIP) is secreted by enteroendocrine K cells in response to nutrient ingestion. The aims of this study were: i) to evaluate the cross-sectional associations between plasma GIP change in response to an oral glucose challenge (as a surrogate of GIP secretion) with obesity-related anthropometric measurements, fasting inflammatory biomarkers, and fasting circulating adipokines; and ii) to evaluate the feasibility of using postprandial plasma GIP as a biomarker of adiposity-related phenotypes in response to starch-based meals.

**Methods:**

Fifty normoglycemic women without obesity (19–32 years) were evaluated with an oral glucose tolerance test (OGTT). A feasibility study was conducted in a subset of eight women to estimate responses to starch-based meals (25 g of starch). Postprandial glycemic-related changes in plasma hormones/metabolites were assessed, and circulating adipokines and inflammatory biomarkers in fasting conditions.

**Results:**

The incremental-GIP change after 2 h OGTT was significantly associated with waist circumference (rho = 0.34; *P* = 0.02), fasting plasma TNF-α (rho = 0.54; *P* = 0.0002), and white blood cell count (rho = 0.39; *P* = 0.008), but not with MCP-1, total adiponectin, leptin, or the free leptin index. A strong inverse association was found between incremental-GIP change and fasting plasma high-molecular-weight (HMW) adiponectin (rho = −0.50; *P* = 0.0004), which remained significant after adjusting for age and body mass index.

**Conclusion:**

An inverse association was found between postprandial GIP levels and circulating HMW-adiponectin levels in humans. This work highlights the suitability of using postprandial plasma GIP as a biomarker for metabolic disturbances of increased adiposity, even in the absence of obesity.

## Introduction

Obesity is a multifactorial disease with systemic implications in glucose homeostasis and a risk factor for type 2 diabetes mellitus (T2DM). A relevant physiological mechanism involved in glucose homeostasis is the incretin effect, which refers to the enhancement of insulin secretion when glucose is administered orally compared to when it is administered intravenously (1). Two intestinal hormones mediate this effect: glucose-dependent insulinotropic peptide (GIP; secreted by enteroendocrine K cells) and glucagon-like peptide 1 (GLP-1; secreted by enteroendocrine L-cells). Both hormones are secreted in response to the ingestion of nutrients, particularly carbohydrates and lipids (2, 3, 4, 5), and are then rapidly degraded by dipeptidyl peptidase-4 (DPP-4) (1). It is reported that the incretin effect is impaired in T2DM, emphasizing the importance of these hormones in glucose homeostasis (2, 6, 7, 8, 9).

The use of GLP-1 agonists and, more recently, dual and triple GLP-1/GIP and GLP-1/GIP/glucagon agonists has been proven to be a useful therapy for treating T2DM and obesity (10). The mechanisms of action of these therapies and the role of endogenous incretins are active areas of research, particularly considering the controversies surrounding agonism/antagonism of GIPR as potential treatments for obesity (11, 12). While GLP-1 has well-documented effects on insulin secretion, delaying gastric emptying, and increasing satiety (1), GIP is a multifaceted hormone that enhances insulin secretion, promotes β-cell survival, regulates bone remodeling, and modulates lipid storage/lipolysis in adipose tissue, among other physiological effects (11, 13). Both GIP and GLP-1 receptors are present in adipose tissue, although GLP-1 receptor expression tends to be low and varies depending on the fat depot and metabolic context. GIP, in particular, has been shown to promote anabolic activity in adipocytes by enhancing the uptake of glucose and free fatty acids (FFAs), especially under hyperinsulinemic conditions, partly through the activation of lipoprotein lipase (14, 15, 16, 17, 18). In experimental studies, GIP was shown to increase triglyceride storage in adipose tissue, a mechanism that may contribute to fat accumulation (11, 18, 19). Interestingly, loss-of-function mutations in the GIP receptor (GIPR) in humans and *Gipr* knockout mice have been linked to resistance to obesity and improved insulin sensitivity (19, 20, 21, 22).

Beyond its function as energy storage, adipose tissue is an active organ that releases a variety of adipokines, including pro-inflammatory mediators, such as tumor necrosis factor-α (TNF-α), leptin, monocyte chemoattractant protein-1 (MCP-1; also known as chemokine C–C motif ligand 2, CCL2), interleukin-1β (IL1β), and interleukin-6 (IL6). It is reported that elevated fasting plasma GIP levels in obesity are associated with increased production of pro-inflammatory cytokines (17), adipocyte lipid deposition, and visceral abdominal fat (23). Leptin is a key adipokine that circulates in proportion to increased body fat in common obesity and is involved in both appetite regulation and chronic mild inflammation associated with obesity (24). Moreover, leptin regulates glucose and lipid metabolism, as demonstrated in rare genetic disorders, such as leptin deficiency or congenital generalized lipodystrophy (25). In contrast, adiponectin and its active form, high-molecular-weight (HMW)-adiponectin, are secreted by adipose tissue and are inversely associated with insulin resistance, adiposity, and circulating pro-inflammatory profiles (23, 26, 27, 28). In this sense, the leptin/adiponectin ratio (LAR) has been proposed as a surrogate marker of insulin resistance and adipose tissue dysfunction (29, 30, 31). On the other hand, a simple and clinically available biomarker of non-specific low-grade inflammation is the white blood cell (WBC) count, which has been linked to an increased risk of type 2 diabetes (32).

Studies in mice have shown that inhibiting GIP signaling enhances plasma levels and adipocyte mRNA expression of adiponectin (33). Despite such a GIP-adiponectin connection reported in experimental models, the relationship between plasma GIP and circulating levels of HMW-adiponectin (the active form of this hormone) remains underexplored in both rodents and humans. A recent extensive review on GIP did not even mention the possible physiological relationship between adiponectin and GIP (11). The aims of this study were: i) to evaluate the association between plasma GIP change in response to an oral glucose challenge (as a surrogate of GIP secretion) with obesity-related anthropometric measurements, fasting inflammatory biomarkers, and fasting circulating adipokines (including total adiponectin and high-molecular-weight-HMW adiponectin); and ii) to evaluate the feasibility and adequacy of using postprandial plasma GIP as a biomarker of adiposity-related phenotypes in response to starch-based meals of highly controlled composition and microstructure.

## Subjects, materials, and methods

### The OGTT study

A cross-sectional study was conducted in 50 non-diabetic, normoglycemic Chilean volunteers without a family history of diabetes, not pregnant (after test of pregnancy), without anemia, with a body mass index (BMI) < 30 kg/m^2^, and aged 19–32 years, as part of a previously published study (30, 34, 35). One week before the OGTT day, a pregnancy test and a hemogram (to rule out anemia) were obtained in all participants in the visit called ‘screening day’. During the second visit, after an overnight fast, participants underwent a standard 2 h oral glucose tolerance test (OGTT) with 75 g of glucose, accompanied by multiple measurements of circulating hormones and metabolites (see the ‘Plasma biochemical and hormone determinations’ section below). An hemogram was again obtained in baseline fasting plasma samples on the day of the OGTT study.

### The feasibility study

A nested small-scale feasibility study was also conducted in a subset of eight volunteers from the OGTT study mentioned above (36). This feasibility study was conducted to estimate the magnitude and variability of postprandial GIP responses after ingesting different starch-based meals (25 g starch +5 g wheat gluten +5.7 g either soy or palm oil) and to assess the acceptance of the meal test. Our research team previously reported all the details on the starch-based meals used in this feasibility study (37). The meals administered to volunteers were extensively characterized in terms of nutrient composition and microstructure through scanning electron microscopy (to assess roughness), polarized light microscopy (to assess gelatinization), and their *in vitro* digestibility properties. As in the OGTT study, multiple circulating hormones/metabolites were measured (see the ‘Plasma biochemical and hormone determinations’ section below). The study protocol was reviewed and approved by the Ethics Committee of the School of Medicine of the Pontificia Universidad Católica de Chile (Santiago, Chile). Written informed consent was signed by all participants of this study.

### Anthropometry

Weight, waist circumference, and height were used to calculate the BMI (kg/m^2^) and the waist-to-height ratio. The conicity index (CI) was used to assess fat distribution and was calculated as CI = waist circumference (cm)/sqrt (weight (kg) × height (m)).

### Plasma biochemical and hormone determinations

Blood samples were collected in EDTA-containing tubes, except for glucose and lactate measurements, which were collected in tubes with the glycolytic inhibitor sodium fluoride and potassium oxalate as anticoagulant. EDTA tubes submitted to subsequent plasma incretins and glucagon measurements were pretreated with antiprotease inhibitors (Sigma-Aldrich, USA, P2714 and Merck-Millipore, Germany, DPP4). Blood samples were processed to obtain plasma, which was then immediately stored at −80°C until analysis.

Plasma glucose and lactate levels (colorimetric methods), insulinemia (electrochemiluminescence), and hemogram (hematocrit, hemoglobin, platelets, and WBC count) were determined in the Central Laboratory (School of Medicine, Pontificia Universidad Católica de Chile). Plasma c-peptide, GIP, GLP-1, glucagon, MCP-1, and TNF-α were quantified using a multiplex ELISA system (MAGPIX, Merck-Millipore). Plasma leptin and adiponectin were measured using radioimmunoassay (RIA) (Linco Research, USA). Plasma soluble leptin receptor (sOB-R) and HMW-adiponectin were measured using standard ELISA immunoassays (R&D Systems, USA). The free leptin index (FLI), the ratio of serum leptin to sOB-R, was calculated as a marker of leptin bioactivity (38). Leptin and C-peptide were measured using both multiplex-MAGPIX and RIA (Supplementary Fig. S1, see the section on Supplementary materials given at the end of the article), showing high correlation and a significant concordance (Lin’s concordance coefficient = 0.38; *P* < 0.0001 for leptin and 0.53; *P* < 0.0001 for C-peptide). Although not entirely satisfactory for analytical laboratory purposes, such RIA-MAGPIX comparisons validate measurements using MAGPIX for association testing purposes. The leptin/adiponectin ratio (LAR) and the leptin/HMW-adiponectin ratio (LAHMWAR) were also calculated as surrogates of insulin resistance (30). Plasma non-esterified fatty acids (NEFAs) were measured with an enzymatic colorimetric method (Wako LabAssay™ NEFA).

In the OGTT study, blood samples were drawn at −15, −5, 15, 30, 60, 90, and 120 min to measure plasma glucose, insulin, C-peptide, and lactate. Measurements from samples at –15 and –5 min were averaged to get the baseline values (‘0 min’). Additional markers, including circulating FFAs, GIP, and GLP-1, were evaluated at baseline (−15 min) and at 120 min post-glucose load. The incremental-GIP change after OGTT or meals was calculated as the difference between plasma GIP levels at 120 min and the baseline value. Common surrogates of insulin secretion and insulin resistance/sensitivity were calculated in the OGTT study (see the list in Supplementary Table S1) (34, 39). In the feasibility study, blood samples were collected at −15 (assigned as the zero time), 15, 30, 60, 90, and 120 min after the start of meal ingestion.

### Statistical analysis and power calculations

In the OGTT study, changes of circulating metabolites and hormones during oral glucose challenges were depicted using areas under the curve and evaluated via the non-parametric sign test for repeated measures. Associations between plasma GIP (basal and incremental levels) and metabolic, hormonal, and anthropometric variables were analyzed using non-parametric Spearman correlations. The leptin/HMW-adiponectin ratio deviated significantly from the normal distribution and was therefore standardized for graphical representation using the rank-based inverse normal distribution. Multiple linear regression models were also used to assess associations, adjusting for potential confounding variables, including age and BMI. A sample size of 50 participants from the original study provides >80% power (at 95% confidence level; *α* = 0.05). to detect correlations of *r* > 0.4 between changes in circulating GIP and adipokine-related continuous variables (40).

In the feasibility study, it is worth noting that sample size/power calculations and statistical inference analyses are typically not included in this type of research; instead, only descriptive statistics are provided (41). In any case, *P*-values of selected statistical tests are shown for illustration purposes. All statistical analyses in the OGTT and feasibility studies were conducted using the STATA 17 package (https://www.stata.com).

## Results

**Figure 1:**
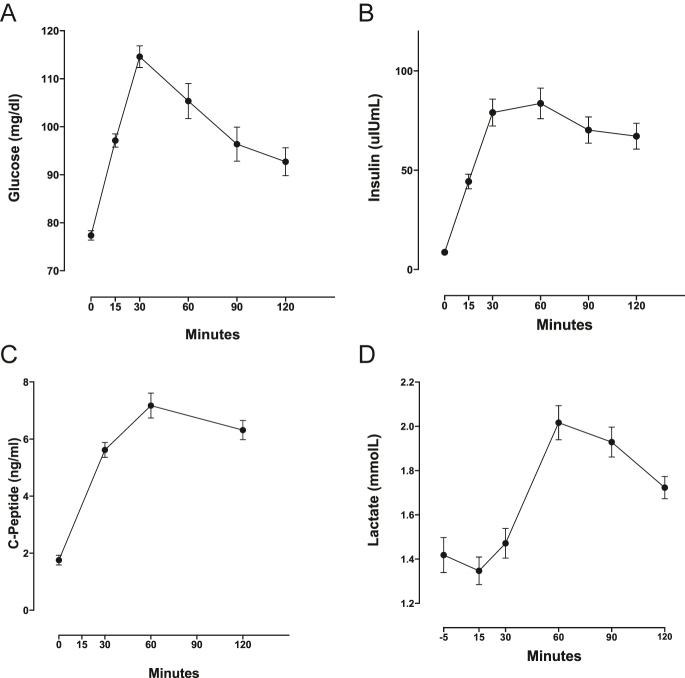
Changes in plasma glucose, insulin, c-peptide, and lactate during OGTT in normoglycemic, non-obese Chilean women. The data are presented as the mean ± standard error of the mean. OGTT: oral glucose tolerance test.

**Figure 2:**
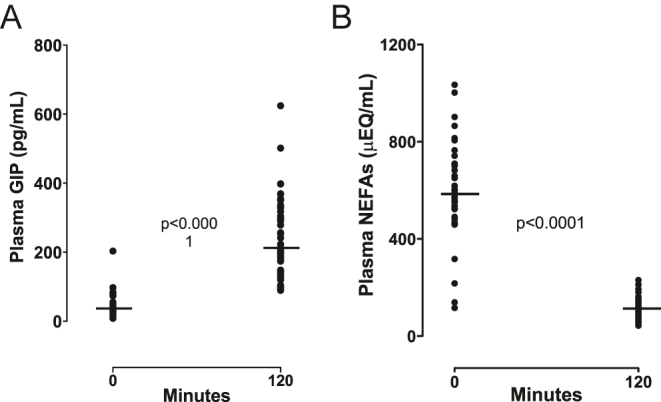
Changes in circulating GIP and NEFAs during OGTT in normoglycemic, non-obese Chilean women. OGTT: oral glucose tolerance test; GIP: glucose-dependent insulinotropic polypeptide; and NEFAs: non-esterified fatty acids. The horizontal bars represent the median values at baseline and after 120 min during OGTT. The horizontal lines represent the medians.

Table 1 summarizes the anthropometric measurements of participants of the OGTT study and their basal circulating levels of adipokine/inflammatory markers. Figure 1 shows the baseline and postprandial plasma levels of glucose (Fig. 1A), insulin (Fig. 1B), C-peptide (Fig. 1C), and lactate (Fig. 1D). Supplementary Table S1 shows insulin secretion/sensitivity indexes calculated from the OGTT data. Figure 2 shows changes in plasma levels of GIP and NEFAs before and after OGTT (baseline −120 min). Plasma GIP levels increased from a baseline mean of 38.2 ± 32.8 pg/mL (mean ± standard deviation) to 244.3 ± 114.7 after 120 min (6.4 mean foldchange; *P* < 0.0001; Fig. 2A). Plasma levels of NEFAs decreased from a baseline mean of 594.4 ± 193.7 μE/L to 108.9 ± 46.6 (81% reduction; *P* < 0.0001) (Fig. 2B). With no exceptions, all volunteers increased their plasma levels of GIP, and all decreased their plasma levels of NEFAs after the OGTT (Fig. 2). Plasma GLP-1 levels at minute 120 returned to their initial baseline levels (Supplementary Fig. S2). A near-significant association was found between incremental-GIP with decremental NEFAs that occurred during OGTT (decremental NEFAs = plasma NEFAs-at-120 min minus NEFAs-basal; rho = 0.30; *P* = 0.055) (Supplementary Fig. S4A).

**Table 1:** Baseline characteristics, anthropometry, and circulating basal adipokine/inflammatory profile of adult Chilean women enrolled in the OGTT study.

Variable	*n*	Mean	SD	P25	P50	P75
Age (years)	50	24.5	3.5	22.0	25.0	27.3
Anthropometry						
Weight (kg)	50	58.9	7.9	53.8	58.0	62.1
Height (m)	50	1.59	0.05	1.56	1.60	1.63
BMI (kg/m^2^)	50	23.1	2.5	21.2	23.1	25.0
Waist (cm)	50	71.2	6.9	66.8	70.0	74.0
Waist-to-height ratio	50	44.7	3.95	41.9	43.6	46.7
CI	50	1.08	0.06	1.03	1.06	1.09
Plasma adipokines and inflammation markers						
MCP1 (pg/mL)	44	53.5	24.8	34.5	47.7	68.4
TNF-α (pg/mL)	42	2.77	1.86	1.70	2.46	3.18
Total adiponectin (ug/mL)	45	11.0	3.99	7.70	10.5	13.5
HMW-adiponectin (ug/mL)	45	3.38	1.33	2.36	3.40	4.26
Leptin (ng/mL)	45	17.3	8.88	10.4	15.9	21.3
Soluble leptin receptor (sOB-R) (ng/mL)	45	25.2	7.7	19.7	24.9	29.7
FLI	45	0.78	0.60	0.40	0.66	1.01
Leptin/adiponectin ratio	45	1.85	1.26	0.90	1.46	2.63
Leptin/HMW-adiponectin ratio	45	7.47	10.6	2.91	4.91	7.77
WBC count (×10^3^/mm^3^)	49	6.47	1.62	5.40	6.10	7.65

HMW, high-molecular weight adiponectin; WBC, white blood cell; CI, conicity index; and FLI, free leptin index.

**Figure 3:**
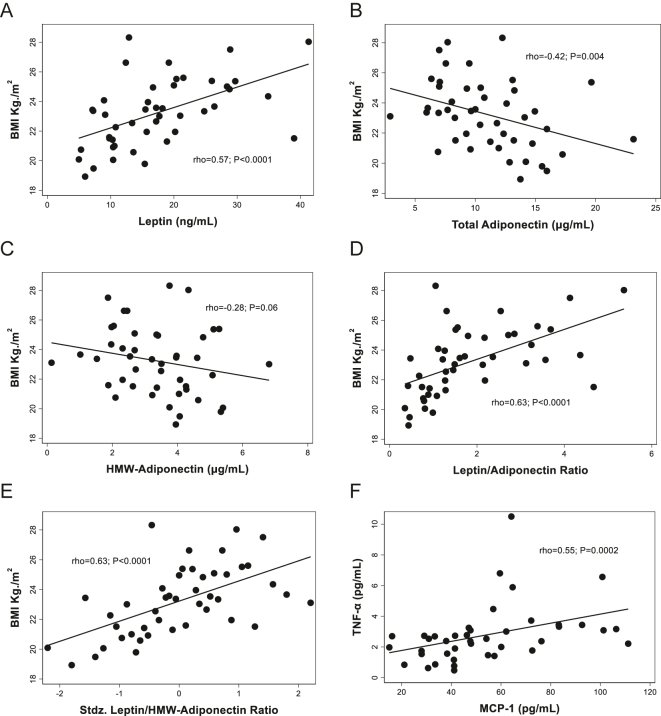
Associations between fasting plasma levels of adipokines and related indexes with BMI in normoglycemic, non-obese Chilean women. BMI: body mass index; TNF-α: tumor necrosis factor-α; and MCP-1: monocyte chemoattractant protein-1 (MCP-1/CCL2).

As expected, BMI was significantly associated with fasting leptin levels (rho = 0.57; *P* < 0.0001; Fig. 3A) while both total adiponectin and HMW-adiponectin showed significantly inverse associations with BMI (Fig. 3B and C). Consequently, leptin/adiponectin ratios, using either total adiponectin or HMW-adiponectin, also showed direct associations with BMI (Fig. 3D and E) and were inversely correlated with the Matsuda insulin sensitivity index (Supplementary Fig. S3). Fasting plasma levels of the proinflammatory adipokines TNF-α and MCP-1 were strongly associated (rho = 0.55; *P* = 0.0002; Fig. 3F). No significant associations were found between fasting plasma levels of GIP with anthropometric, biochemical, hormonal (including adipokines), or insulin secretion/sensitivity indexes derived from OGTT (such as those shown in Supplementary Table S1).

**Figure 4:**
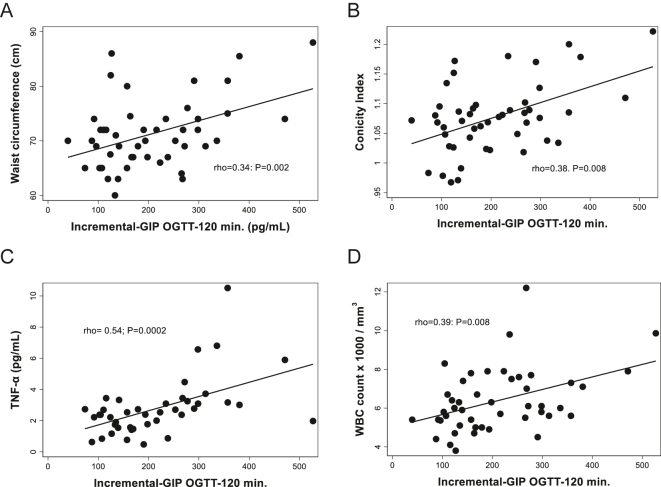
Associations between incremental-GIP during OGTT with anthropometric measurements and selected plasma adipokines and biomarkers in normoglycemic, non-obese Chilean women. Oral glucose tolerance test. Incremental-GIP: 2 h minus basal plasma levels of GIP during OGTT. TNF-α: tumor necrosis factor-α and WBC: white blood cell. OGTT.

We found that incremental-GIP during OGTT was directly associated with waist circumference (rho = 0.34; *P* = 0.02; Fig. 4A), conicity index (rho = 0.4; *P* = 0.01; Fig. 4B), waist-to-height ratio (rho = 0.29; *P* = 0.049; Supplementary Fig. S4B), and fasting plasma TNF-α levels (rho = 0.54; *P* = 0.0002; Fig. 4C). A significant association was also found between incremental-GIP and WBC count, measured both on the OGTT day (rho = 0.39; *P* = 0.008; Fig. 4D). Moreover, this significant association was replicated when the WBC count was measured on the screening day (rho = 0.32; *P* = 0.03). No significant association was found between the incremental-GIP and plasma levels of MCP-1, plasma leptin levels, leptin soluble receptor, or the FLI (Supplementary Fig. S4C and Supplementary Fig. S4F).

**Figure 5:**
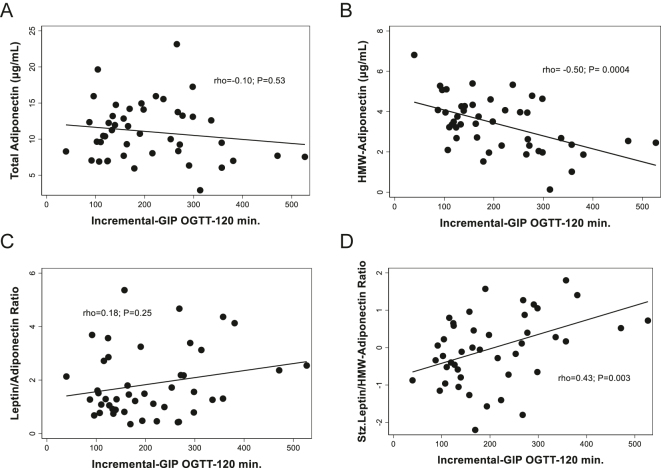
Associations between incremental-GIP during OGTT with plasma adiponectin, plasma HMW-adiponectin, and leptin/adiponectin ratios in normoglycemic, non-obese Chilean women. OGTT: oral glucose tolerance test. Incremental-GIP-120 min: 2 h minus basal plasma levels of GIP during OGTT. HMW-adiponectin: high-molecular-weight adiponectin; the prefix Stz. stands for standardized (see Statistical Methods).

The incremental-GIP after OGTT showed no association with fasting plasma total adiponectin (Fig. 5A). However, a significant inverse correlation was found between incremental-GIP and fasting plasma HMW-adiponectin, with rho = −0.50; *P* = 0.0004 (Fig. 5B). Such an association was still significant after correction for multiple comparisons. In a simple regression model, incremental-GIP accounted for 26.4% of the variability in HMW adiponectin (*P* < 0.0001), and this association was still significant after adjusting for age and BMI (*P* = 0.003). No association was found between incremental-GIP and the leptin/adiponectin ratio (Fig. 5C), while a significant correlation was detected between incremental-GIP and the standardized leptin/HMW adiponectin ratio (rho = 0.43; *P* = 0.003) (Fig. 5D).

**Figure 6:**
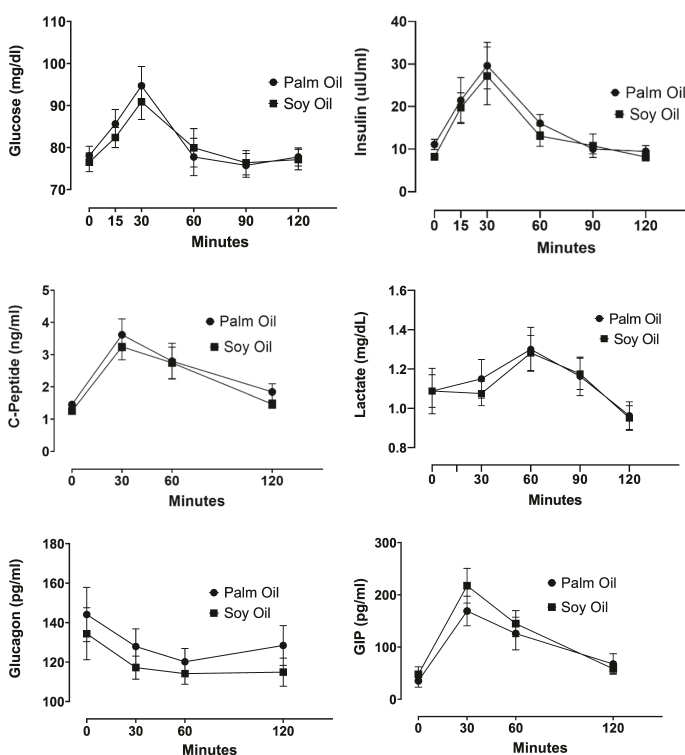
Feasibility study for changes in plasma glucose, insulin, c-peptide, glucagon, and GIP after ingestion of starch-based meals in normoglycemic, non-obese Chilean women. OGTT: oral glucose tolerance test and GIP: glucose-dependent insulinotropic peptide. The data are presented as the mean ± standard error of the mean. No *P*-values are reported in the feasibility study (see text).

In the feasibility study, a subset of *n* = 8 volunteers from the OGTT study was selected to assess GIP responses against consuming two starch-based meals with either palm or soy oil. The age range of this group was 20–30 years, and the BMI range was 18.9–24.4 Kg/m^2^. Supplementary Table S2 shows the sensory evaluation of the meals used in this study (starch with soybean oil versus palm oil). We observed the expected changes in plasma metabolites and hormones (including GIP) using both meals. Plasma GIP levels increased postprandially, reaching a maximum at 30 min, with no significant differences between the two meals. Expected postprandial changes were also found for glucose, insulin, c-peptide, lactate, and glucagon (Fig. 6). As in the OGTT study, GLP-1 plasma levels were similar at baseline and after 120 min in both meal tests. Integrating data from both meals, we calculated the incremental-GIP value at minute 30 for each participant by averaging the two incremental-GIP-30 min values obtained after ingesting both meals. Compared to the baseline, the rise of GIP at minute 30 was 4.7-fold (*P*-value = 0.0039, one-sided test), while the increase at minute 120 was 1.5-fold (*P*-value = 0.14, one-sided). Interestingly, the averaged incremental-GIP-30 min obtained in the feasibility study also showed a noteworthy high inverse correlation with fasting plasma levels of HMW-adiponectin (rho = −0.61; *P* = 0.08) (Supplementary Fig. S4B), replicating what was observed previously in the OGTT Study (Fig. 5B). As mentioned in the ‘Subjects, materials, and methods’ section, *P*-values in the feasibility study are reported only for illustration purposes.

## Discussion

The present study reveals a significant inverse association between GIP postprandial elevations following an OGTT (the incremental-GIP, defined as plasma 2 h minus basal levels of this hormone) and fasting levels of circulating HMW-adiponectin, a key adipokine linked to increased insulin sensitivity. This finding is particularly relevant, given that HMW-adiponectin is considered the biologically active form of adiponectin, a key adipokine associated with protection against insulin resistance and obesity-related inflammation (42). Notably, most studies only measure total circulating levels of adiponectin, rather than HMW-adiponectin. Then, our study suggests that chronically elevated GIP postprandial levels, using incremental-GIP as a surrogate, may negatively impact the protective actions of adiponectin by reducing the plasma levels of its active variant (HMW-adiponectin).

The significant direct correlation between the incremental GIP and visceral adiposity indexes found in our study (waist circumference, waist-to-height ratio, and conicity index) is partially concordant with a previous report that found a direct association between stimulated GIP and an unhealthy pattern of fat distribution, but only in men (43). Moreover, we also found that postprandial incremental-GIP was positively associated with biomarkers of low-grade inflammation, such as plasma TNF-α and WBC count, but not with MCP-1 (18, 44, 45, 46). To our knowledge, this is the first study to report a direct association between incremental GIP and WBC count, a nonspecific biomarker of inflammation. Interestingly, elevated fasting plasma levels of GIP have been associated with higher cardiovascular events and mortality in epidemiologic studies (47). In contrast to the significant associations detected in our study between incremental-GIP and anthropometric/metabolic indices, no associations were found between fasting plasma GIP and visceral obesity, insulin resistance indexes, plasma adipokines, or low-grade inflammation.

Our findings, which link the increase in postprandial GIP secretion with reductions in systemic plasma levels of HMW-adiponectin, align with previous research in rodent models, where inhibition of GIP signaling enhanced circulating adiponectin levels and improved metabolic outcomes, particularly under high-fat dietary conditions. It is reported that the absence of GIP receptor signaling in *Gipr*^−/−^ mice resulted in increased plasma adiponectin and its expression in adipose tissue (33). While these animal studies suggest that suppressing GIP may increase adiponectin expression, our results indicate that higher postprandial GIP secretion is associated with lower HMW-adiponectin levels. In addition, our results provide support for using the leptin/adiponectin ratio as an index of adipose tissue dysfunction, showing a strong association with BMI in participants without obesity. In this sense, the positive association between the incremental-GIP and the leptin/HMW-adiponectin ratio, a marker of insulin resistance and adipocyte dysfunction, also reinforces the hypothesis that elevated GIP may impair adiponectin-mediated pathways, potentially contributing to insulin resistance and metabolic dysfunction (30). Given the established links between GIP, obesity, inflammation, and insulin resistance, our findings reinforce the notion that endogenous GIP plays a relevant role in the metabolic dysregulations associated with increased adiposity (43).

It has been reported that GIP may promote adipogenesis under hyperinsulinemic conditions and exhibit lipolytic effects under hypoinsulinemia (43, 48, 49). In OGTT and oral meal challenges, interpreting the dynamic associations between changes in plasma GIP during an OGTT and concurrent variations in hormones/metabolites (glucose, insulin, lactate, NEFAs, and GLP-1, among others) is problematic, given the multiple concurrent effects mediated by these hormones/metabolites, particularly insulin (50). For this reason, the impact of GIP elevations on insulin secretion, resistance/sensitivity, and lipid deposition was typically evaluated in the literature under hyperglycemic clamps to remove the effects of insulin and glucose changes on metabolism (48). Despite this limitation of our OGTT data, we found a trend for a direct significant association between an increase in postprandial GIP (incremental-GIP) and a reduction in plasma NEFAs levels (decremental-NEFAs) during OGTT. This observation is consistent with the effect of endogenous postprandial GIP in favoring lipid deposition in adipose tissue under hyperinsulinemia (18, 43, 48, 49). In support of this idea, *Gipr*^−/−^ knock-out mice are resistant to obesity on high-fat diets (22), and low-frequency loss-of-function mutations in the human GIPR gene have been linked to leanness and impaired bone strength (51). However, it is also reported that GIPR expression in adipose tissue appears to be downregulated in the subcutaneous adipose tissue of obese patients and correlates negatively with BMI, potentially disrupting the GIP/GIPR axis (52). Given the growing interest in GIP biology, its pleiotropic effects, and the therapeutic applications of incretin analogs, further research is needed to determine whether endogenous GIP, in spite of its short action in circulation before inactivation, may interact with the effects of dual GIP/GLP-1 agonists or triple GIP/GLP-1/glucagon agonists used in obesity (11).

Several studies have evaluated plasma adiponectin levels during OGTT (53, 54), with no reported changes in plasma total adiponectin. However, it is reported that there is a selective decline in HMW-adiponectin levels during OGTT in male subjects with normal glucose tolerance or impaired fasting glucose (53). In this sense, it has been shown that HMW-adiponectin levels are selectively reduced under hyperinsulinemic conditions during glucose clamps, although this effect appears to be prevented by hyperglycemia (55, 56). The assessment of relative changes in HMW-adiponectin levels during an OGTT is even more complex, given the concurrent variations in plasma insulin, glucose, incretins, and other metabolites/hormones, as mentioned above. Our study focused on fasting plasma adiponectin (either total or HMW-adiponectin) and shows that women with higher GIP plasma postprandial levels are associated with lower HMW-adiponectin constitutive plasma levels in fasting conditions.

A feasibility study was also conducted to assess the magnitude of postprandial GIP responses and the acceptability of starch-based meals with highly controlled composition. Rather than focusing on inferential statistical analysis, feasibility studies concentrate on determining the most appropriate intervention (in this study, starch-based meal tests) to assess the magnitude and variability of the outcomes (in this case, GIP postprandial responses). Based on the OGTT and feasibility studies, we propose that the incremental-GIP is a suitable and useful biomarker for metabolic disturbances associated with increased adiposity. Using starch-based meals with around 25 grams of carbohydrates generates a much lower response in GIP and is less sustained (returned to near basal levels at 120 min) compared to OGTT. Several studies suggest that the peak of plasma GIP concentrations occurs between 30 and 60 min after the OGTT, with levels maintained relatively unchanged until 120 min (57). Herein, we propose that the incremental-GIP at 120 min adequately captures the GIP dynamics during OGTT, while the incremental-GIP at 30 min is more adequate in the meal challenge test. Similar to findings in other studies, we observed that baseline plasma levels of GLP-1 show similar concentrations after 120 min of the OGTT (57). Interestingly, the observation derived from the OGTT study regarding the inverse association between incremental-GIP and HMW-adiponectin was replicated, or even amplified, using data from the feasibility study. A limitation of our OGTT study is related to its cross-sectional design, lack of determination of the phase of the menstrual cycle, small sample size, and limited statistical power to detect associations. However, these limitations are counterbalanced by a homogeneous sample in terms of age, sex, and cardiometabolic status, which strengthens the internal validity of our study.

In conclusion, this study reports for the first time an inverse relationship between postprandial GIP plasma elevations and fasting circulating HMW-adiponectin levels in humans, suggesting a potential role of GIP in modulating adiponectin actions. Plasma postprandial GIP changes are also directly associated with fat distribution and fasting pro-inflammatory markers in our study. This work highlights the suitability of postprandial plasma GIP as a biomarker of metabolic disturbances of increased adiposity, even in the absence of obesity.

## Supplementary materials



## Declaration of interest

The authors declare that there is no conflict of interest that could be perceived as prejudicing the impartiality of the work reported.

## Funding

This work was supported by FONDECYT grant 1241497 (Principal Investigator: Javier Parada) and DIDEMUC grant PMD 03-24 (School of Medicine, MSc Nutrition, Pontificia Universidad Católica de Chile, Santiago, Chile) awarded to Isidora Salvatierra.

## Institutional review board statement

All participants provided signed informed consent. The project was reviewed and approved by the Scientific Research Committee of the Pontificia Universidad Católica de Chile (ID 230914002). The study was conducted in accordance with the Helsinki guidelines and regulations.

## Ethical approval statement

This study was approved by the Scientific Ethics Committee of Health Sciences of the Pontificia Universidad Católica de Chile (CEC-Salud UC) (Resolution No. 012793, Ordinary Session No. 08, May 30, 2024).
