# Supplementary material for: Association between plasma glucose-dependent insulinotropic polypeptide and active adiponectin in normoglycemic women
Source: Endocr Connect. 2026 Jan 13;15(1):e250804. doi: 10.1530/EC-25-0804 (PMC12811713; doi:10.1530/EC-25-0804)
Supplement: Supplementary file 1 [file supplementary_materials.pdf]

SUPPLEMENTARY MATERIAL

Association between glucose-dependent insulinotropic peptide plasma levels after an oral glucose load and high molecular weight adiponectin in normoglycemic women

Table S1. Insulin secretion and resistance/sensitivity indexes in normoglycemic, non-obese Chilean women enrolled in the OGTT study

	Type	n	Mean	SD	P25	P50	P75
**Indexes based on fasting measures**							
HOMA-S	ISens	50	0.75	0.46	0.49	0.67	0.82
Adipo-IR	IR	42	49.1	23.5	35.9	46.4	58.5
QUICKI	ISens	50	0.36	0.02	0.35	0.36	0.37
R-QUICKI	ISens	42	0.40	0.04	0.38	0.39	0.42
TG/HDL index	IR	50	1.70	1.32	1.04	1.35	1.83
TyG index	IR	49	4.43	0.24	4.30	4.42	4.57
TyG-BMI index	IR	49	103.8	12.8	94.3	104.7	112.1
C-peptide index (CPI; basal)	ISecr	46	2.23	1.37	1.66	1.92	2.27
**OGTT-based indexes**							
Matsuda index	ISens	50	5.65	3.05	3.50	4.92	6.90
Hepatic Insulin Resistance Index (HIRI)	IR	48	3.94	2.38	2.41	3.24	4.93
Muscle Insulin Sensitivity Index (MISI)	ISens	49	1.73	0.78	1.00	1.47	2.28
C-peptide index-120 (120 min. OGTT)	ISecr	46	6.96	2.22	5.88	6.92	8.40
Insulinogenic index (OGTT: 0-120 min)	ISecr	50	0.66	0.30	0.45	0.62	0.80
ISSI-2 Oral Disposition Index	ISecr*	49	3.20	1.22	2.35	2.90	3.72

OGTT: Oral Glucose Tolerance Test. HOMA: Homeostasis Model Assessment ; HOMA-S: HOMA-Insulin sensitivity = 1/(fasting insulin (μUI/mL) × fasting glucose (mg/dL)/405) = 1/HOMA-IR. This index is the inverse of the HOMA-IR index (HOMA-S = 1/HOMA-IR). QUICKI: quantitative insulin sensitivity check index= 1 / [log(Fasting Insulinemia) + log(Fasting Glycemia)]; R-QUICKI: Revised QUICKI= 1/[log(fasting glucose) + log(fasting insulin) + log(NEFA)]. Adipose-IR: Adipose-Insulin Resistance index (plasma fasting insulin × free fatty acids). TyG index = Triglyceride-Glucose index. TyG-BMI index = Triglyceride-Glucose-BMI index. CPI: C-peptide index = [(C-peptide concentration in ng/mL) / (Plasma glucose concentration in mg/dL)] x 100. ISSI-2: ISSI-2: Insulin Secretion-Sensitivity index-2 = Matsuda index x Insulinogenic index (0-120 min); ISens: Insulin Sensitivity; IR: Insulin Re; Resistance; ISecr: Insulin Secretion; ISecr*: β-cell compensation (Insulin Secretion adjusted by systemic insulin sensitivity; oral disposition index)

Table S2. Sensory evaluation of the meals used in the feasibility study (starch with soybean oil versus palm oil.

Attribute	Visual Scale (0-10)	Score (Range) with Soybean Oil	Score (Range) with Palm Oil
Flavor	Very bad – Very tasty	0	0
Smell	Unpleasant – Pleasant	-	-
Texture	Unpleasant – Pleasant	0	3-4
Satiety	Not satisfied – Very satisfied	6-7	5-7

Note: The scores represent sensory perceptions based on a 0-10 visual analog scale.

A low score was reported by participants for the food items in the flavor and smell (absence of odor) attributes, with a somewhat better perception of texture in the food containing palm oil compared to soybean oil. A high score of satiety was achieved with both meals. It is worth noting that these starch-based meals were not designed to be pleasant and tasty, but to serve as an acceptable "model food" with tight control over composition and microstructure in studies of postprandial hormonal/metabolic response. Participants easily consumed the meal within a maximum of 5 minutes, chewing approximately 15 times each bite before swallowing, along with 150 mL of water

Figure S1. Scatter charts, Lin´s concordance coefficients, and Bland-Altman plots for the concordance of measurements of fasting plasma leptin and C-peptide using either radioimmune assay (RIA) or multiplex MAGPIX

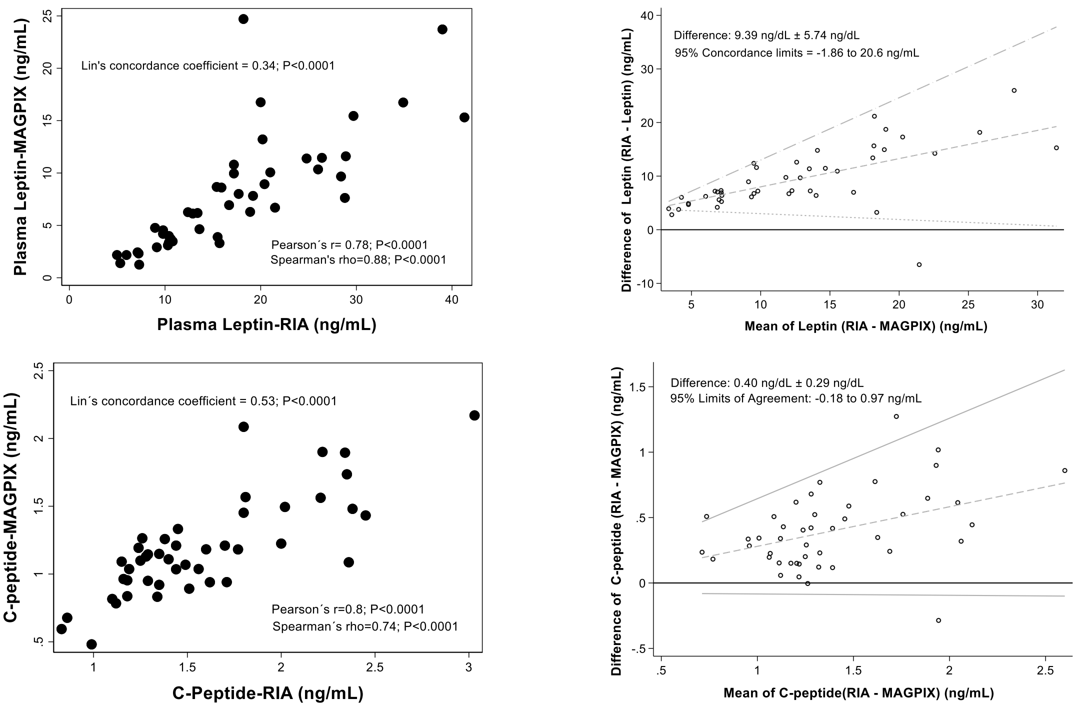

Figure S2. Changes in median circulating active GLP-1 before-after OGTT in normoglycemic, non-obese Chilean women.

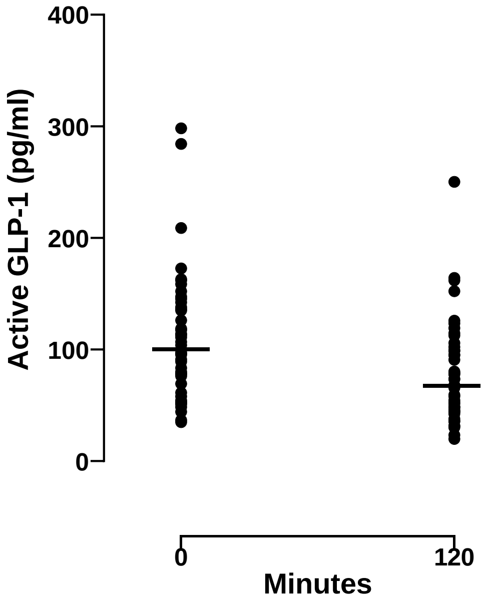

OGTT: Oral Glucose Tolerance Test; GLP-1: Glucagon-like Peptide-1; Horizontal bars represent the median values at baseline and after 120 min. during OGTT

Figure S3. Association between plasma leptin/adiponectin ratio and Matsuda index in normoglycemic, non-obese Chilean women

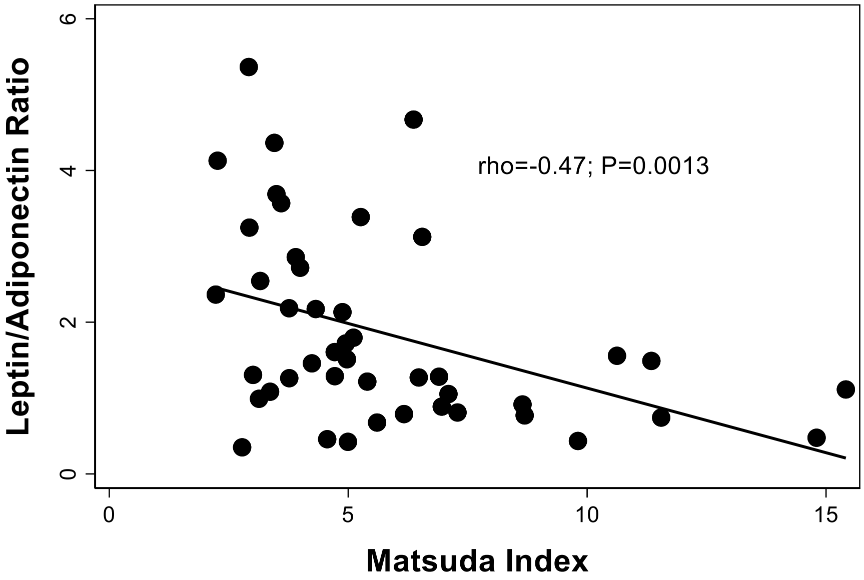

Figure S4. Associations between incremental-GIP (plasma GIP-basal minus GIP-120min) with selected variables in normoglycemic, non-obese adult Chilean women of the OGTT study.

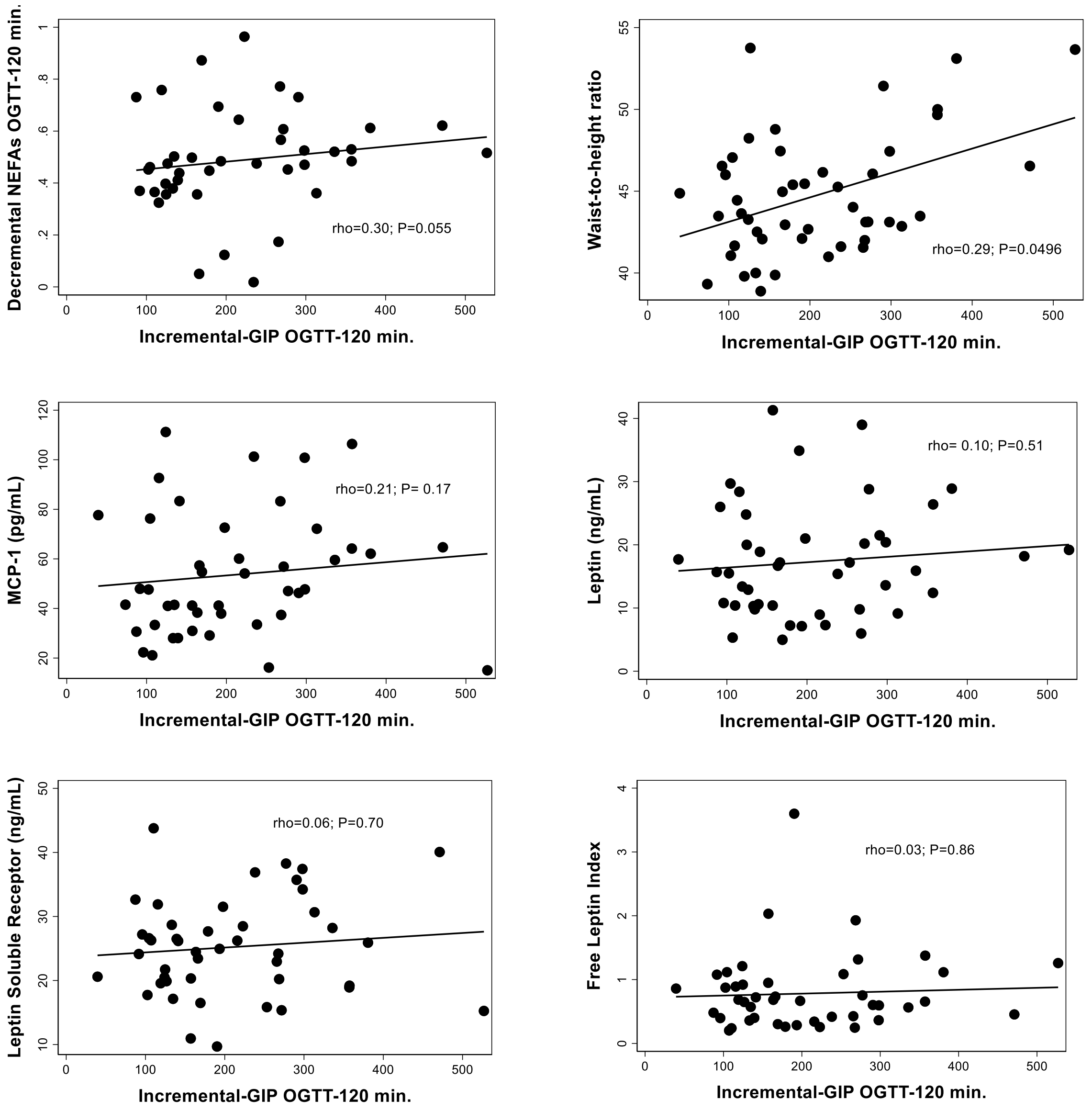

OGTT: Oral Glucose Tolerance Test. Incremental-GIP: 2-hour minus basal plasma levels of GIP during OGTT. Decremental NEFAs: Basal NEFAs – 120 min. NEFAs (μEq/mL). All biochemical variables were measured in plasma.

Figure S5. Association between incremental-GIP (plasma GIP-basal minus GIP-120min) and basal plasma Total Adiponectin and HMW-adiponectin in the feasibility study.

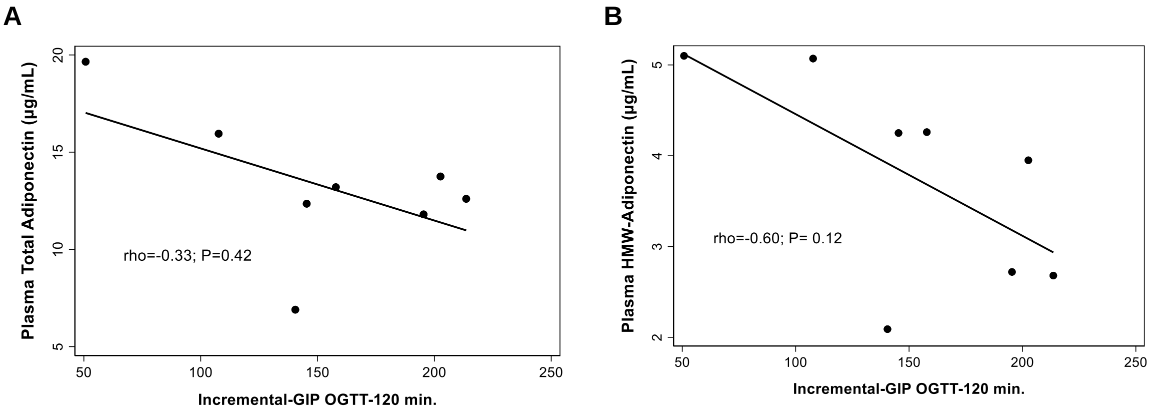

OGTT: Oral Glucose Tolerance Test. Incremental-GIP: 2-hour minus basal plasma levels of GIP during OGTT.
